# Supplementary material for: Mutational Landscape of Bladder Cancer in Mexican Patients: KMT2D Mutations and chr11q15.5 Amplifications Are Associated with Muscle Invasion
Source: Int J Mol Sci. 2023 Jan 6;24(2):1092. doi: 10.3390/ijms24021092 (PMC9866210; doi:10.3390/ijms24021092)
Supplement: Supplementary file 1 [file ijms-24-01092-s001.zip › ijms-2017914-supplementary.pdf]

Mutational landscape of bladder cancer in Mexican patients: *KMT2D* mutations and chr11q15.5 amplifications are associated with muscle invasion

María D. Pérez-Montiel[1], Dennis Cerrato-Izaguirre[2,3], Yesennia Sánchez-Pérez[3], Jose Diaz-Chavez[3], Carlo Cortés-González[3], Jairo A. Rubio[3], Miguel Jiménez-Ríos[4], Luis A. Herrera[5], Anna Scavuzzo[4], Abelardo Meneses-García[7], Ricardo Hernández-Martínez[3], Felipe Vaca-Paniagua[8], Andrea Ramírez[9], Alicia Orozco[9], David Cantú-de-León[6], Diddier Prada[3,10].

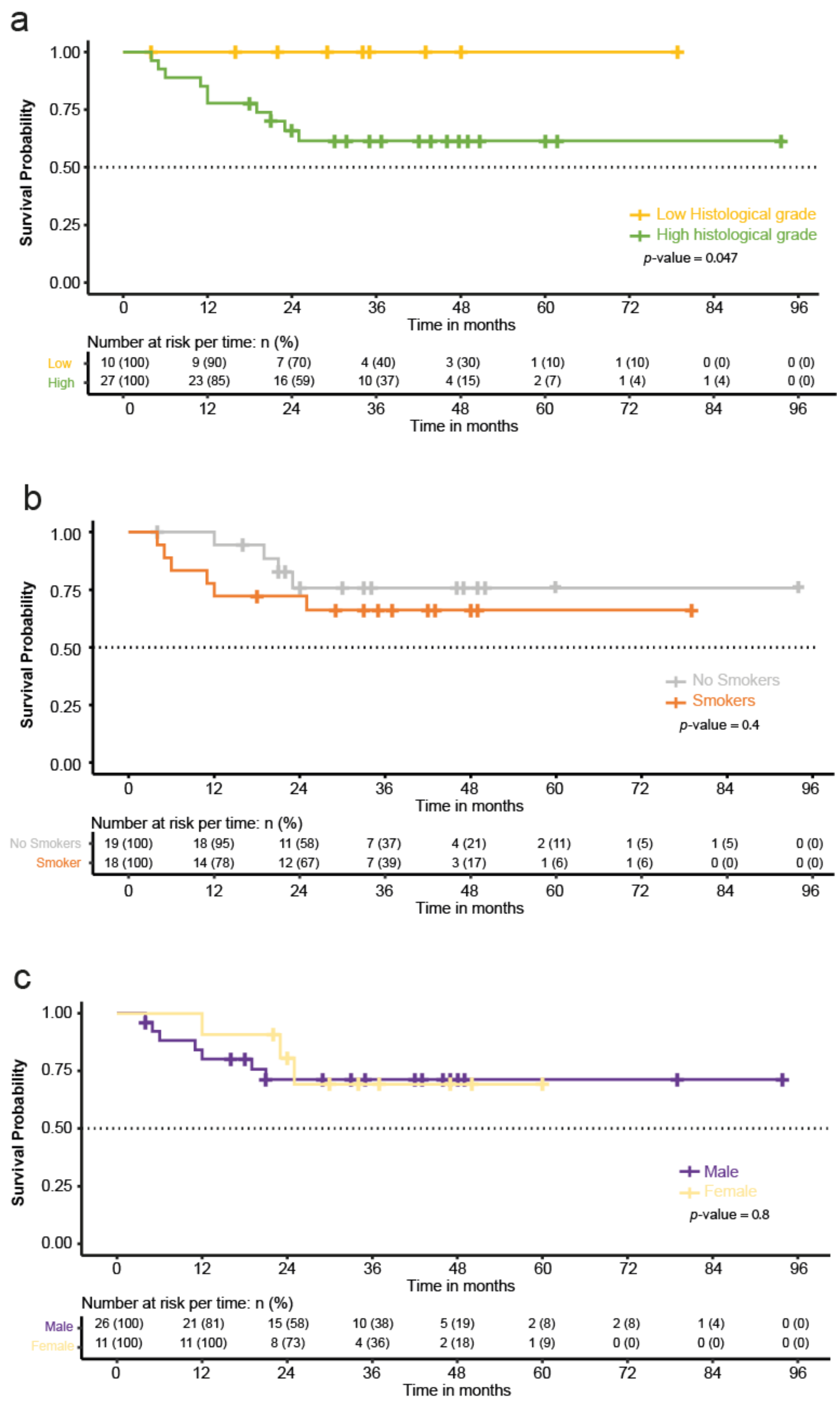

Supplementary Figure S1. Overall survival of the patients with bladder cancer with other clinical features. a) Kaplan-Meier curve of the overall survival by histological grade of histological, b) smokers and c) sex. No significant differences exist using the Log Rank test.

Supplementary Table S1. Multivariate sensitivity Cox proportional hazard models for the influence of muscular invasion on mortality adjusted by clinical-pathological characteristics of patients with bladder cancer attended at the National Cancer Institute - Mexico between 2012 and 2021 (N=37).

	Muscular invasion	Adjusted by histological grade	Adjusted by smoking status	Adjusted by sex	Adjusted by age
Muscular invasion HR (95%CI)	13.11 (1.66, 103.60)	6.83 (0.86, 54.02)	15.67 (1.95, 125.74)	13.26 (1.67, 104.92)	12.37 (1.56, 97.97)
Histological grade HR (95%CI)		9.771 (0.00, Inf)			
Smoking status HR (95%CI)			2.54 (0.71, 9.15)		
Sex HR (95%CI)				0.77 (0.19, 2.98)	
Age HR (95%CI)					4.39 (0.92, 20.81)

HR= Hazard ratio, CI= confidence interval

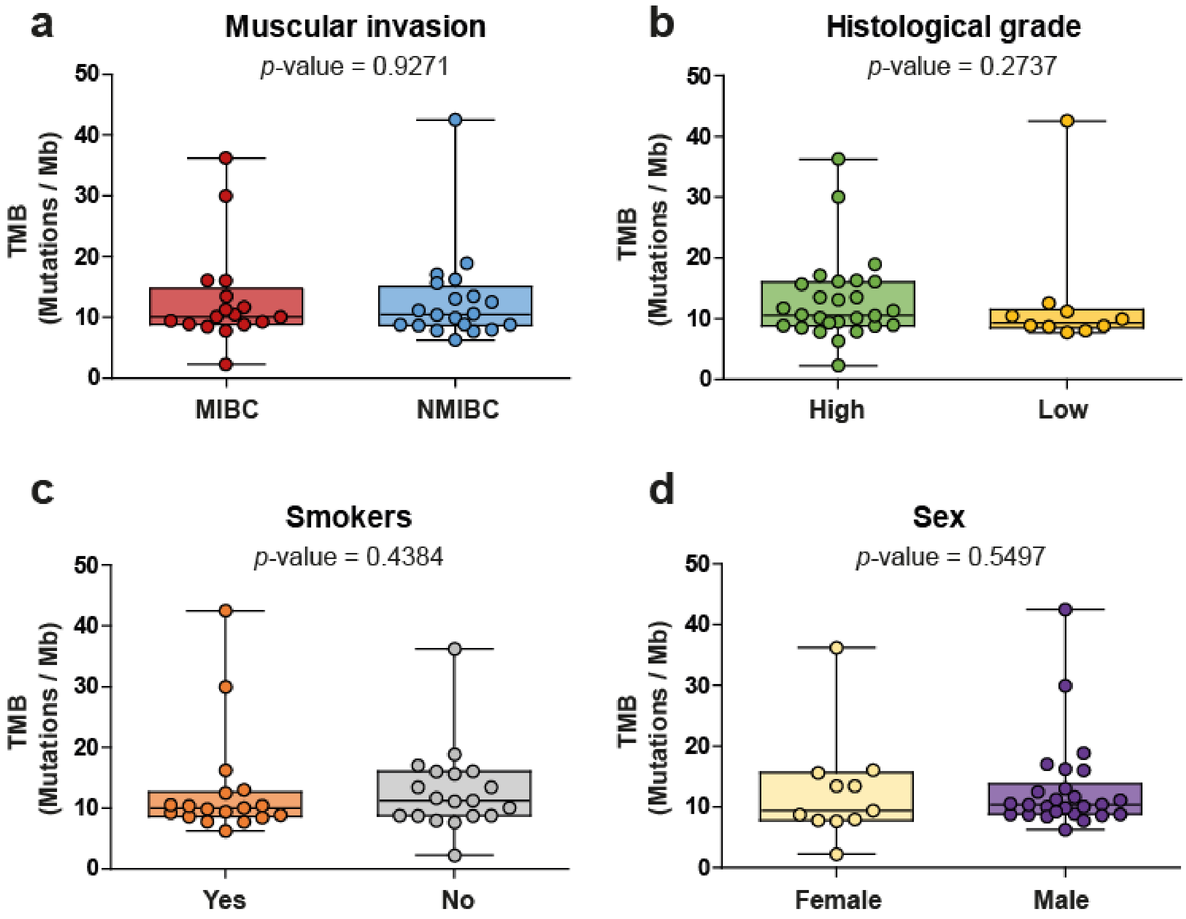

Supplementary Figure S2: Relationship between TMB value and clinical features. a) Comparison of TMB value with the muscular invasion. In red when the muscular invasion is present (Muscle Invasive Bladder Cancer). In blue without muscular invasion (Non-Muscle Invasive Bladder Cancer) b) Comparison of TMB value with histological grade. c) Comparison of TMB value if the people are smokers or not. D) Comparison of TMB value by sex. Each dot shows individual data. *p*-values were calculated using the Mann-Whitney U test.

Supplementary Table S2. Cox proportional hazard model for the influence of muscular invasion on mortality adjusted by the presence of *TP53* mutation, *KMT2D* mutation, chr11p15.5 amplification, or chr19p13.11-q12 amplification in patients with bladder cancer attended at the National Cancer Institute - Mexico between 2012 and 2021 (N=37).

	Muscular invasion	Adjusted by TMB	Adjusted by TP53 mutation	Adjusted by KMT2D mutation	Adjusted by chr11p15.5 amplification	Adjusted by chr19p13.11-q12 amplification
Muscular invasion HR (95%CI)	13.11 (1.66, 103.60)	13.03 (1.64, 103.31)	8.09 (0.95, 68.78)	11.66 (1.46, 92.94)	14.03 (1.70, 115.51)	16.70 (2.03, 137.26)
TMB status HR (95%CI)		0.93 (0.26, 3.31)				
TP53 mutation HR (95%CI)			3.58 (0.71, 17.93)			
KMT2D mutation HR (95%CI)				2.97 (0.85, 10.35)		
chr11p15.5 amplification HR (95%CI)					0.88 (0.42, 1.86)	
chr19p13.11-q12 amplification HR (95%CI)						0.42 (0.11, 1.54)

HR= Hazard ratio, CI= confidence interval, TMB = Tumor mutational burden

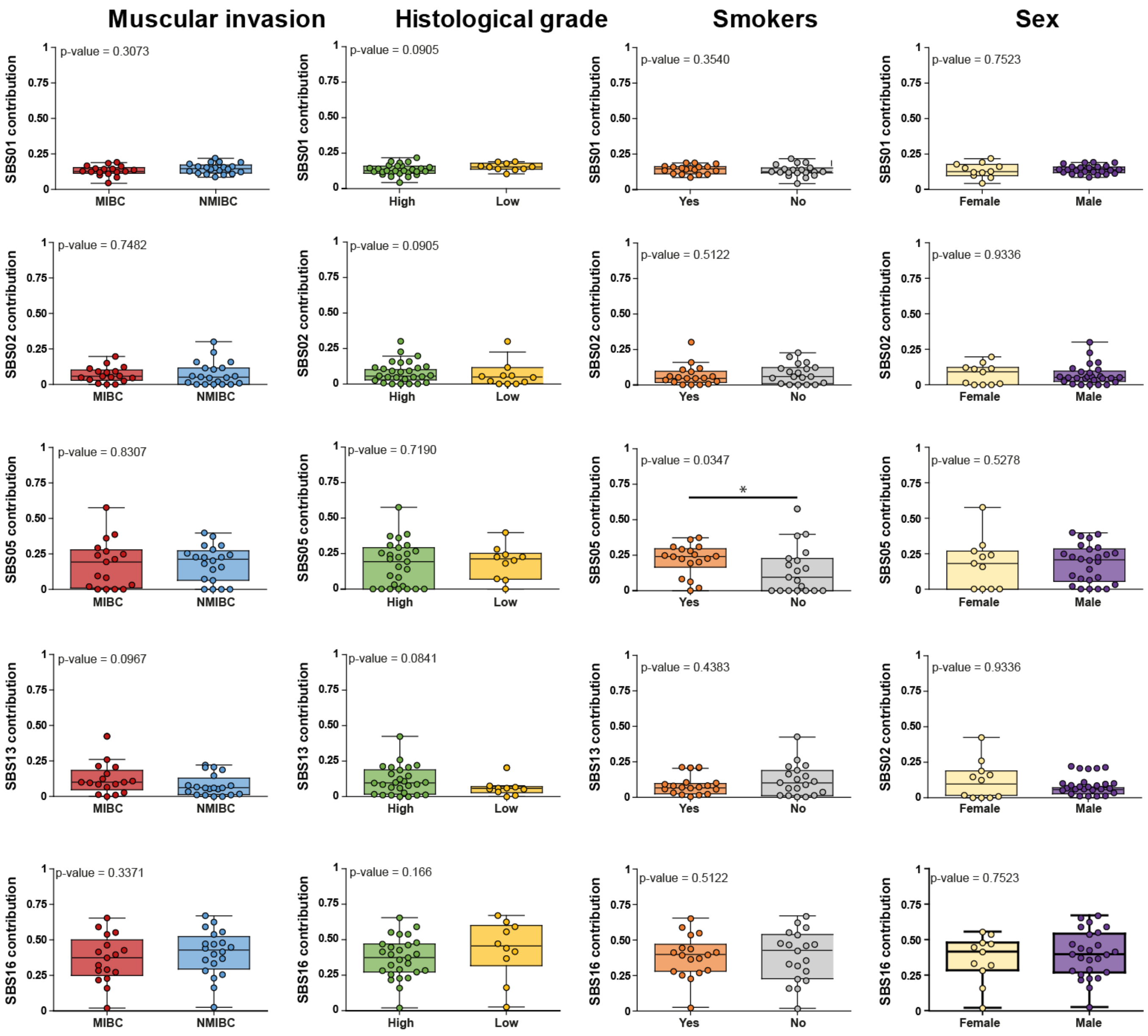

Supplementary Figure S3: Association between mutational signature contribution and clinical characteristics of patients with bladder cancer of the SBS01, SBS02, SBS05, SBS13, and SBS016. Watch the mutation SB05 is statistically significant with previous reports. Each dot shows individual data. *p*-values were calculated using the Mann-Whitney U test.
